# A Spelling Paradigm With an Added Red Dot Improved the P300 Speller System Performance

**DOI:** 10.3389/fninf.2020.589169

**Published:** 2020-12-03

**Authors:** Yan Wu, Weiwei Zhou, Zhaohua Lu, Qi Li

**Affiliations:** School of Computer Science and Technology, Changchun University of Science and Technology, Changchun, China

**Keywords:** brain-computer interface (BCI), P300 speller, visuospatial information, spelling paradigm, event-related potential

## Abstract

The traditional P300 speller system uses the flashing row or column spelling paradigm. However, the classification accuracy and information transfer rate of the P300 speller are not adequate for real-world application. To improve the performance of the P300 speller, we devised a new spelling paradigm in which the flashing row or column of a virtual character matrix is covered by a translucent green circle with a red dot in either the upper or lower half (GC-RD spelling paradigm). We compared the event-related potential (ERP) waveforms with a control paradigm (GC spelling paradigm), in which the flashing row or column of a virtual character matrix was covered by a translucent green circle only. Our experimental results showed that the amplitude of P3a at the parietal area and P3b at the frontal–central–parietal areas evoked by the GC-RD paradigm were significantly greater than those induced by the GC paradigm. Higher classification accuracy and information transmission rates were also obtained in the GC-RD system. Our results indicated that the added red dots increased attention and visuospatial information, resulting in an amplitude increase in both P3a and P3b, thereby improving the performance of the P300 speller system.

## Introduction

Brain–computer interface (BCI) systems allow people to communicate without using their muscles, which provides a direct communication pathway for patients with severe amyotrophic lateral sclerosis (ALS) and other locked-in syndromes (LIS) (Sellers and Donchin, 2006; Kubler and Birbaumer, 2008). An ERP is a response of the brain to an external stimulus, which is generally used to implement a BCI system. The P300 is an ERP component generated from the observation of a rare or odd event and manifests as a positive waveform appearing around 300 ms after presentation of the stimulus (Bernat et al., 2001). In 1988, Farwell and Donchin described a BCI system, known as the P300 speller, which allows the patient to spell characters by detecting the P300 potential (Farwell and Donchin, 1988). In the P300 speller, a 6 × 6 matrix of characters is displayed on a screen, and the rows and columns of the matrix are intensified (flashed) one after another in a pseudo-random order. When users wish to output a target character, they need to only focus on the desired target character. When the row or column containing the target character is intensified, which has a one-sixth probability and constitutes a rare event, a P300 potential is elicited. Thus, the target character is determined by the row and the column that elicited a P300 potential. Several studies have attempted to improve the spelling accuracy and speed of the P300 speller. However, its performance is still unable to meet the requirements of a real-world application (Kaufmann et al., 2011; Aya et al., 2018; Philip and George, 2020; Xu et al., 2020).

Eliciting larger amplitudes of ERP such as P300, to improve the performance of character recognition, is a key direction for optimizing BCIs (Aya et al., 2018; Xiao et al., 2019). Previous studies have indicated that focusing attention on external stimuli improves the processing of visual information in the nervous system and can significantly modulate the visual stimulus response (Posner, 1980; Mangun, 1995). Further, the resource quantity expended on concentrating attention directly affects the excitability of brain activity and resulting features of the evoked waveform (Berti, 2016). Lakey et al. (2011) reported that heightening subjects' attention with a short session of mindfulness meditation can elicit larger P300 amplitude. Additionally, researchers have shown that there is a reciprocal relation between the concentration of attentional resources and the scope or size of the attentional focus (Eriksen and Yeh, 1985; Xu et al., 2018). When attention is paid to a small spatial scope, the stimulus is allocated more visual processing resources, resulting in a greater ability of the brain to process and discriminate the stimulus (Rincover and Ducharme, 1987).

Stimuli containing spatial information can elicit larger ERP amplitudes than those without. A previous study found that a stimulus located above or below the central fixation point elicited a larger P300 amplitude than one located at the central fixation point (Abramov et al., 2017). Several studies reported that when the appearance of the target was predictable, subjective efforts in perceptual processing and attention orientation were small, resulting in the reduction of the target P300 amplitude (Sutton et al., 1965; Hugdahl and Nordby, 1994). Therefore, we speculated that changing the visuospatial location to reduce the probability of the target appearance could also increase the P300 amplitude.

Green has been shown to be a color that helps the perceivers maintain attention on a task (Xia et al., 2018). Studies have investigated the combining of characters or stimulus images with green backgrounds to modify spelling paradigms and found that they improved not only the comfort level of subjects but also the performance of the P300 speller (Li et al., 2015; Lu et al., 2019).

In the present study, we proposed a new spelling paradigm to attract more attention from subjects and increase visuospatial information, in which the flashing row or column of a virtual character matrix was overlaid with a translucent green circle in which a red dot was positioned in either the upper or lower half (GC-RD spelling paradigm). The red dot resulted in a smaller focus scope, and its appearance in either the upper or lower half of the green circle reduced the probability of its manifestation. The control spelling paradigm was that the flashing row or column of the virtual character matrix was covered by a translucent green circle only (GC spelling paradigm). We compared ERP waveforms and the spelling performances of the P300 speller between the two paradigms to verify whether the GC-RD spelling paradigm would improve the performance of the P300 speller system.

## Materials and Methods

### Participants

Eleven college student volunteers (two female and nine male; mean age, 20 ± 2 years old) participated in the study. Participants signed their written informed consent after receiving a full explanation of the purpose and requirements of the study. All participants were right handed and had normal or corrected-to-normal vision. Two of the participants had previously participated in a similar experiment, while the others had no prior BCI experience. The study was approved by the ethics committee of Changchun University of Science and Technology.

### The Spelling Paradigm

**Figure 1:**
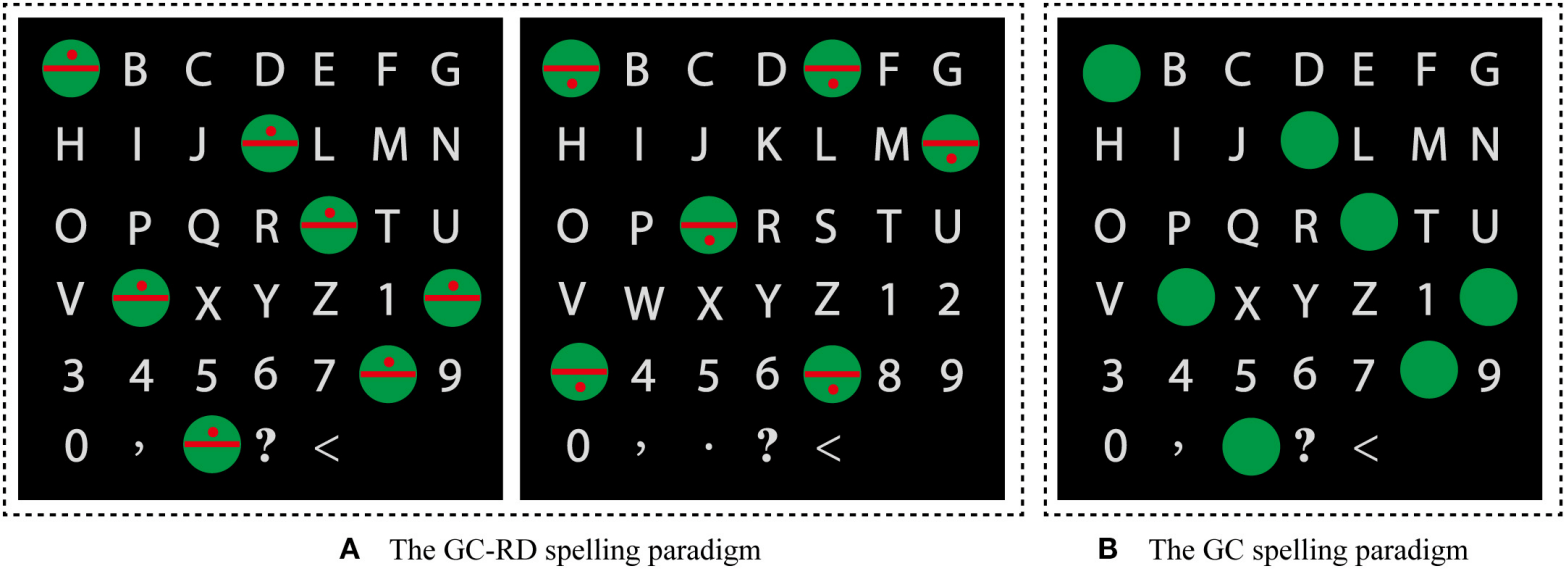
The spelling paradigms. **(A)** The GC-RD spelling paradigm: the red dot appears in the upper (left picture) or lower half (right picture) of the green circle. **(B)** The GC spelling paradigm.

**Figure 2:**
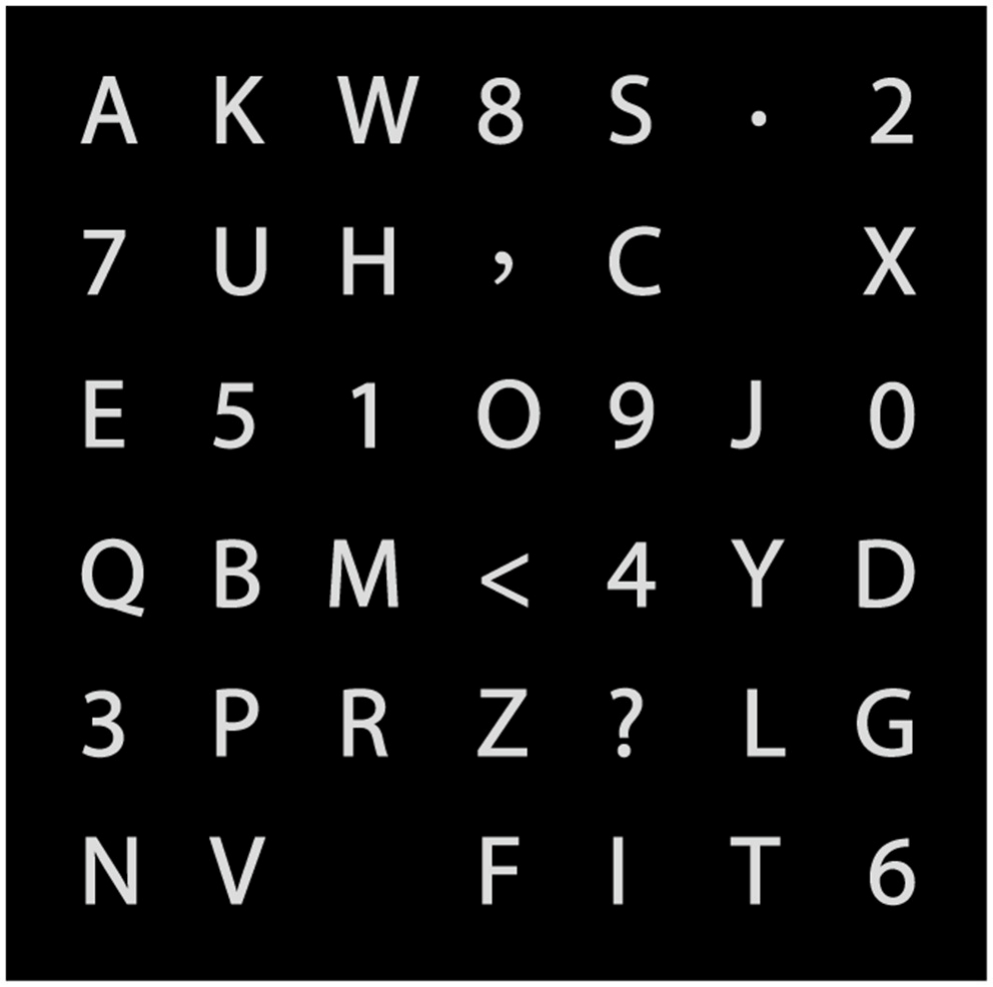
The 6 × 7 virtual character matrix.

In both the GC-RD and GC paradigms, a 6 × 7 character matrix with 26 letters, 10 numerals (0–9), and four symbols is presented on a monitor (Figure 1). The size of each character is 1.2° × 1.2° (1.5 × 1.5 cm), and the distance between each character is 3.5° × 2.5° (4.5 × 3 cm). To mitigate the problem of adjacency flashing, we pseudo-randomly intensified a set of characters (six or seven) that scattered as far away as possible. The intensified characters were selected according to the rows and columns of a virtual 6 × 7 character matrix as shown in Figure 2.

In the GC-RD paradigm, characters are covered by green circles with a red dot while intensified. The red dot appears in the upper (Figure 1A, left) or lower (Figure 1A, right) half of the green circle. The position of the red dots is the same for all intensified stimuli in each flash. The GC paradigm is similar to the GC-RD paradigm but without the red dot (Figure 1B). The interstimulus interval (ISI) was 250 ms, in which each character was covered by a green circle with (GC-RD paradigm) or without (GC paradigm) a red dot for 200 ms and then reverted to a gray character for 50 ms.

### Procedure

**Figure 3:**
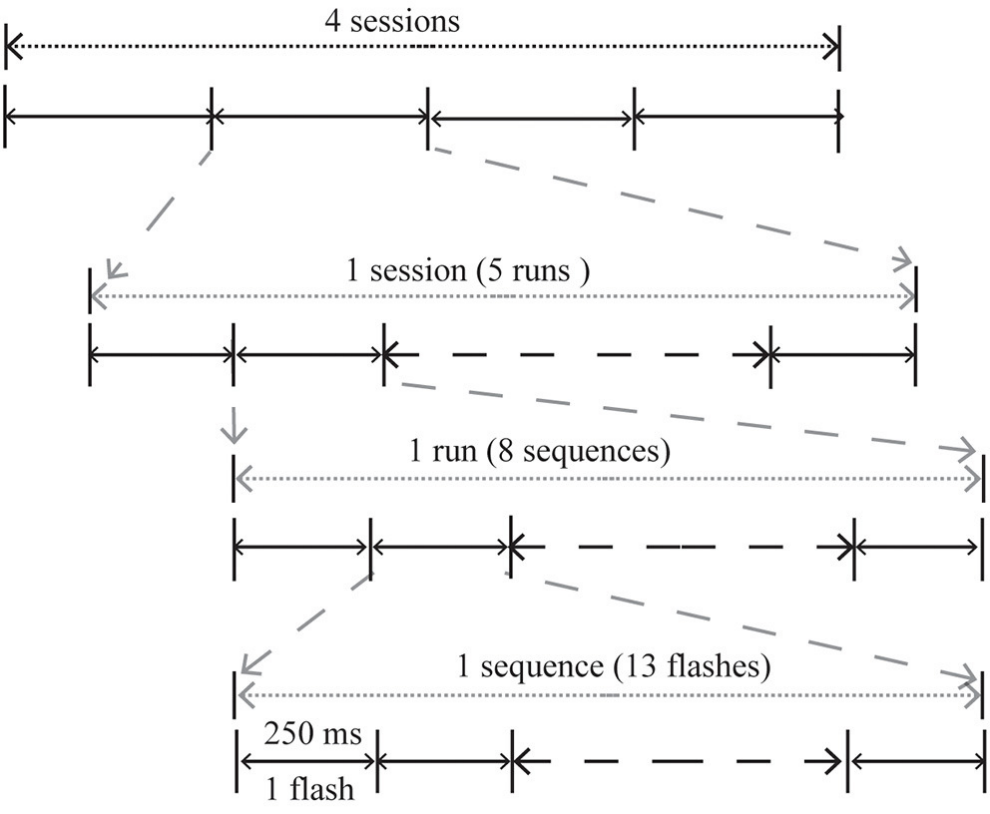
The procedure of the two experiments.

The study was conducted in a dimly lit, sound-attenuated, and electrically shielded room. Participants sat ~90 cm in front of a monitor. Each participant participated in two experiments: Exp. 1 (GC-RD spelling paradigm) and Exp. 2 (GC spelling paradigm). Each experiment consisted of four sessions in which the subjects were required to output four words with five different characters; each session included five runs to output the five characters. Eight sessions of two experiments were conducted in a pseudo-random order to avoid learning effects. Thirteen flashes corresponding to the six rows and seven columns were defined as a sequence. In each run, the sequence was repeated eight times. Thus, each run consisted of 104 flashes of row or column to output a target character (Figure 3). In Exp. 1, the 13 flashes in a sequence comprised six occasions when the red dot was in the upper half of the green circle and seven when it was in the lower half.

During the experiments, subjects were instructed to avoid unnecessary movement including blinking, to pay attention to the target character, and to silently count the number of target character flashes. In Exp. 1, participants were specifically told to concentrate their attention on the red dot rather than on the whole green circle. Subjects were allowed to take a 5-min break between two sessions.

### Data Acquisition

Electroencephalograph (EEG) data from 14 channels (F3, Fz, F4, C3, Cz, C4, P3, Pz, P4, P7, P8, O1, Oz, and O2) were recorded by a SynAmps2 EEG amplifier (SynAmps 2, NeuroScan Inc., and Abbotsford, Australia) with the left mastoid as the ground and the right mastoid as reference. Horizontal eye movements were measured by placing two horizontal electrooculogram (HEOG) electrodes at the corners of the left and right eyes. Two vertical electrooculogram (VEOG) electrodes were placed ~1 cm above and 1 cm below the left eye to record vertical eye movements. In data preprocessing, the EEG signals that were contaminated by EOG were corrected using a regression analysis algorithm. The impedance of these electrodes was kept below 5 kΩ. All data were digitized at a rate of 250 Hz.

### Data Processing and Analysis

The raw EEG data were filtered between 0.1 and 30 Hz using a third-order Butterworth band pass filter. The EEG signals were then divided into epochs from 100 ms before the onset of each flashing to 800 ms after the onset, and baseline corrections were made against −100–0 ms. Amplitudes of the P3a and P3b components in the two time windows at 14 of the electrode channels were analyzed with a 2 (spelling paradigms: GC-RD vs. GC) × 14 (electrode channels) repeated measures analysis of variance (ANOVA). The Greenhouse–Geisser Epsilon correction was applied to adjust the degrees of freedom of the F ratios, if necessary. Because a greater difference between target and nontarget trials simplifies their classification, the difference waveforms (ERP_Target_ – ERP_nontarget_) for both experiments were obtained by subtracting ERP waveforms elicited by nontarget trials from those elicited by target trials.

### Classification Scheme

The EEG data were classified using Bayesian linear discriminant analysis (BLDA). BLDA is an extension of Fisher's linear discriminant analysis, which avoids overfitting due to high-dimensional and possibly noisy datasets (Jin et al., 2010, 2015). The details of the algorithm have been published (Lei et al., 2009), and many studies have shown that BLDA achieves perfect results in P300 detection (Jin et al., 2012, 2014). We used fourfold cross-validation to calculate individual spelling accuracy, successively choosing one of the four sessions as the test set and the remaining three as the training sets, thus obtaining the accuracy of the test set. Individual accuracy was obtained by averaging the four results for each participant.

### Information Transfer Rate

The information transmission rate (ITR) was first described by Wolpaw et al. (1998) and is used to evaluate the communication performance of a BCI system. ITR (bit/min) refers to the amount of information that can be transmitted per minute, with the calculation formula as follows:

(1) $$\begin{array}{r} B=\log_{2}N+P\log_{2}P+\left(1-P\right)\log_{2}\frac{1-P}{N-1} \end{array}$$

(2) $$\begin{array}{r} ITR\left(bits/min\right)=B\times \frac{60}{T} \end{array}$$

where *N* is the number of possible choices within a sequence, and *P* is the target identification accuracy. *B* (bit/trial) is the number of bits per trial transmission, and *T* (seconds/character) is the time needed to output each character.

In addition, because of low signal-to-noise ratios, we calculated and compared the classification accuracy and ITR with different sequence numbers to investigate the effects of changing the number of averaged sequences.

## Results

**Figure 4:**
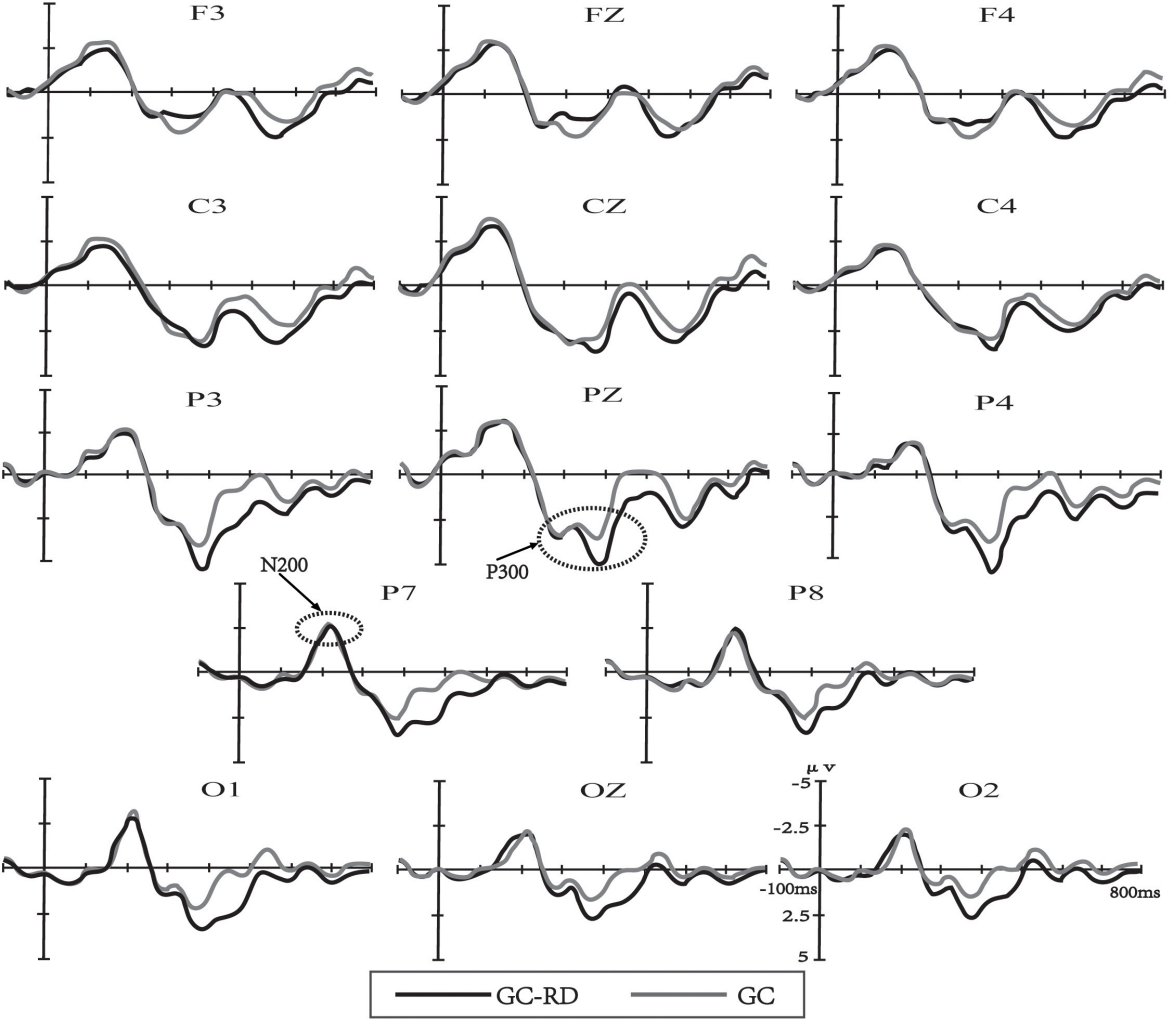
Superimposed grand average waveforms elicited by target trials from 11 students in the GC-RD and GC spelling paradigms. The P300 potential (Pz) and N200 component (P7) are circled.

Figure 4 shows the grand-average waveform elicited by target trials from 11 students in two spelling paradigms. Positive deflections were clearly observed at the central area (C3, CZ, and C4), parietal area (P7, P3, PZ, P4, and P8), and occipital area (O1, OZ, and O2) in both paradigms, indicating the P300 potential ERP component (Polich, 2007). In addition, a clear negative waveform was observed around 200 ms at the bilateral temporal area (P7 and P8) and occipital area (O1, Oz, and O2) in both paradigms; this is likely to be the N200 component (Reza et al., 2007).

The GC-RD spelling paradigm stimulus elicited a higher P300 potential than the GC spelling paradigm at the central, parietal, and occipital areas (Figure 4). A biphasic positive component between 250 and 500 ms was visible with two peaks: the first peak between 250 and 350 ms and the second peak between 350 and 450 ms. The first positive deflection may be P3a potential and the second may be P3b potential (Berti, 2016).

**Figure 5:**
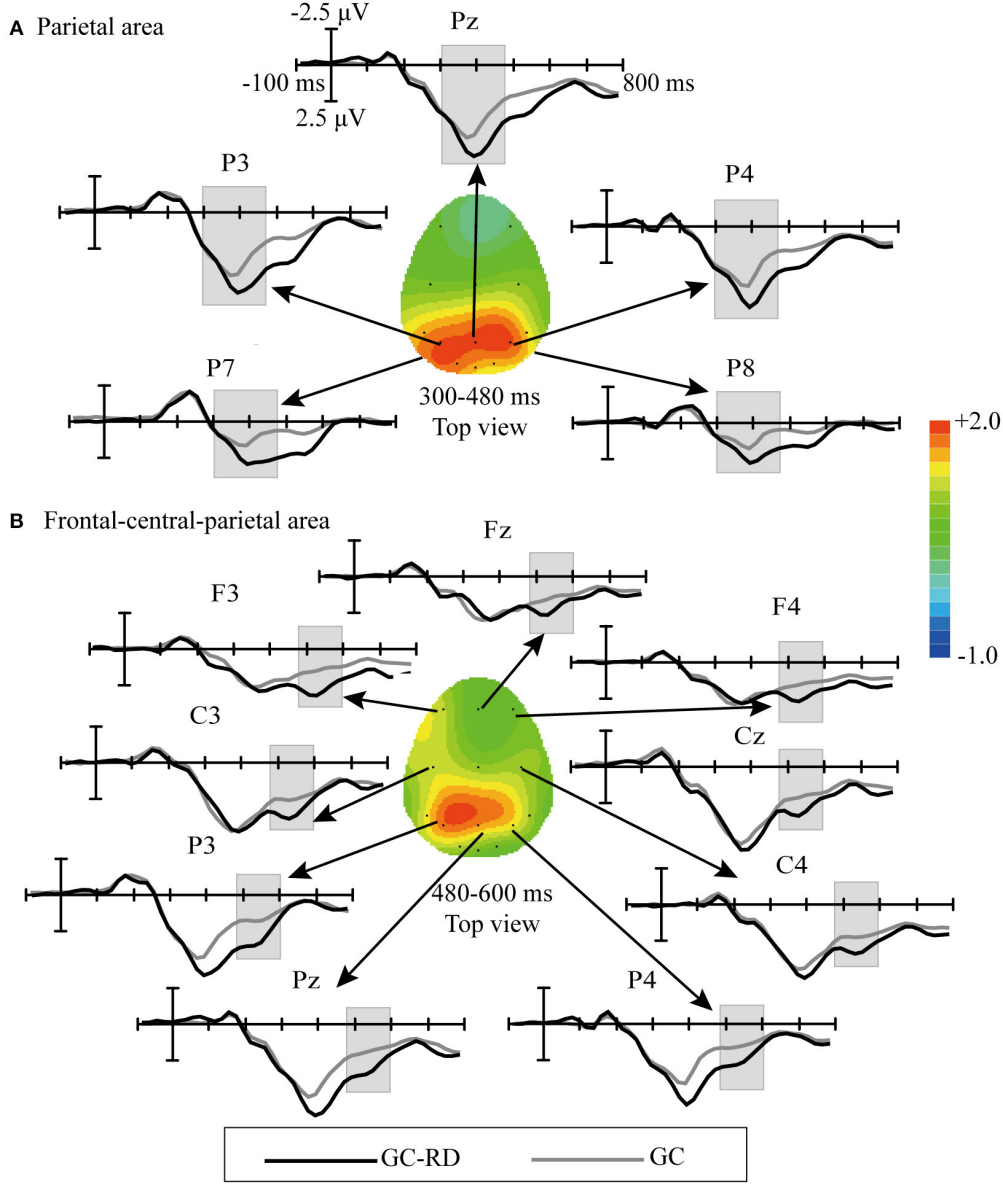
Comparison of difference waveforms of event-related potential (ERP) by subtracting the ERPs of the GC spelling paradigm from those of the GC-RD spelling paradigm (ERP_Target_ – ERP_Nontarget_) and scalp topographies for double difference waveforms obtained by subtracting the (ERP_Target_ – ERP_Nontarget_) waveforms for the GC spelling paradigm from those of the GC-RD spelling paradigm. **(A)** Parietal area at 300–480 ms; **(B)** Frontal–central–parietal areas at 480–600 ms.

Analysis of the difference waveforms (ERP_Target_ – ERP_Nontarget_) between the GC-RD and GC spelling paradigms showed significant differences for P3a in 300–480 ms at P7, P3, Pz, P4, and P8 [*F*_(1, 10)_ = 25.5111, *P* = 0.001] and for P3b in 480–600 ms at F3, Fz, F4, C3, Cz, C4, P3, Pz, and P4 [*F*_(1, 10)_ = 6.654, *P* = 0.03]. The significant difference amplitudes of P3a were mainly at the parietal areas (Figure 5A), while the significant difference amplitudes of P3b were at the frontal–central–parietal areas (Figure 5B).

Based on the ERP analysis, we intercepted 160–688 ms from the −100–800 ms data for feature extraction from 11 channels (F3, Fz, F4, C3, Cz, C4, P3, Pz, P4, P7, and P8) to reduce the computational time, in which the significant differences of the ERP waveforms were observed (Li et al., 2015). The intercepted EEG data were downsampled from 250 to 62.5 Hz by selecting every fourth sample from the filtered EEG signals. This decreased the number of waveform points to 33. Therefore, the size of the feature vector was 33 × 11, with 11 denoting the number of electrodes and 33 denoting the number of sample points in each flashing.

**Figure 6:**
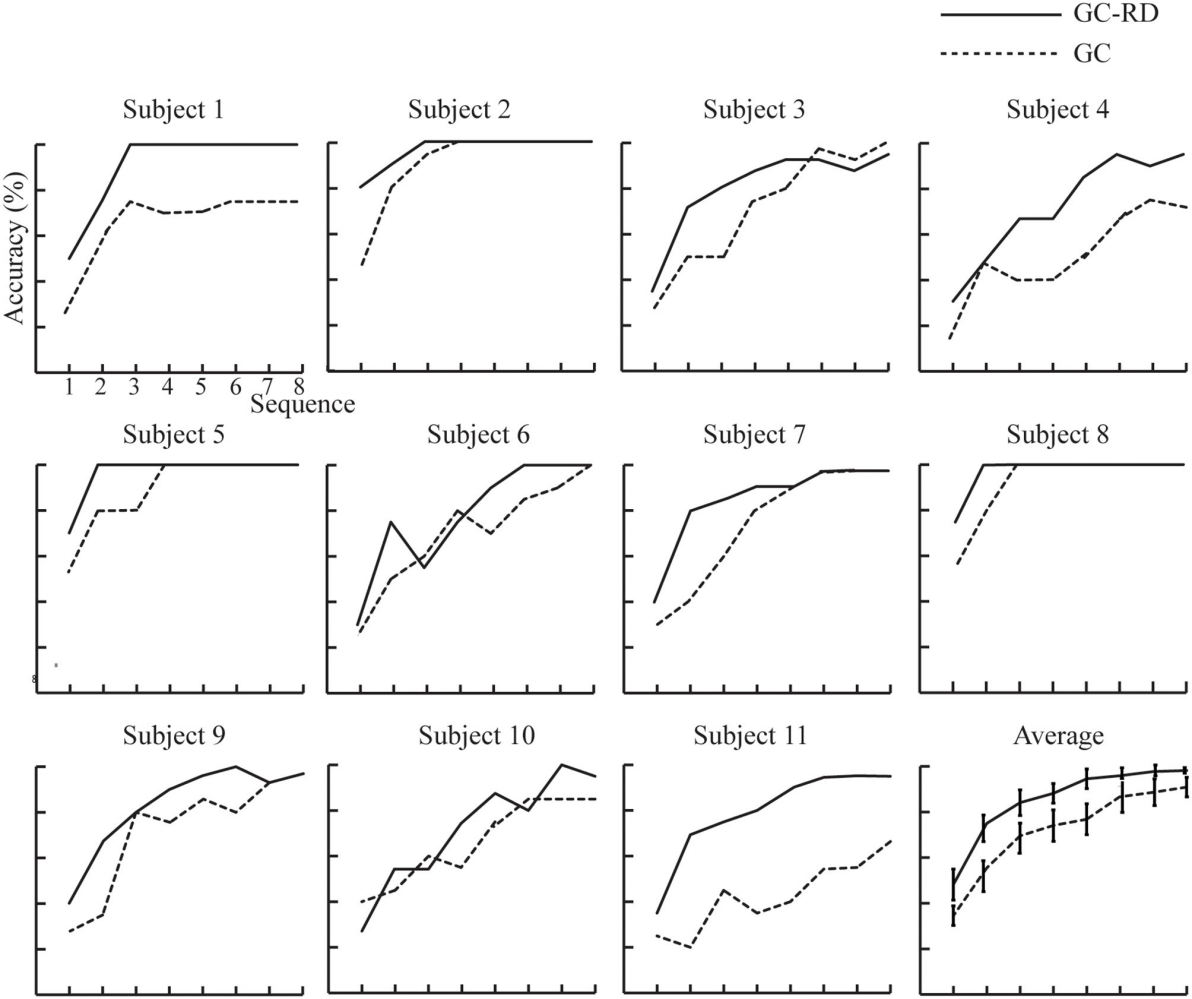
Individual and average accuracies of the P300 speller from 11 subjects with different sequence number in the GC-RD and GC spelling paradigms.

Figure 6 shows the individual and average accuracies; accuracy increased as sequence number increased for both spelling paradigms. The average classification accuracy of the GC-RD spelling paradigm was higher than that of the GC spelling paradigm at all sequence numbers. In the GC-RD spelling paradigm, the classification accuracy of five subjects (subjects 1, 2, 5, 6, and 8) reached 100% with an average sequence of 3.2. Before statistically comparing classification accuracy and ITR, we verified that the data were normally distributed by a one-sample Kolmogorov–Smirnov test (Jin et al., 2014). A paired sample *t*-test was then conducted to compare the accuracy between GC and GC-RD spelling paradigms at each sequence. Results of the *t*-tests showed that the GC-RD spelling paradigm was significantly more accurate than the GC spelling paradigm at sequences 1–7 (Table 1).

**Table 1:** Results of paired sample *t*-tests comparing accuracy between the GC-RD and GC spelling paradigm at each sequence time.

	**Sequence**							
	1^**^	2^**^	3^**^	4^**^	5^*^	6^*^	7^*^	**8**
GC	33.64 (±3.99)	53.18 (±6.00)	67.73 (±5.89)	73.18 (±6.88)	79.09 (±6.10)	85 (±4.57)	86.82 (±4.17)	89.55 (±4.07)
GC-RD	47.27 (±5.74)	75 (±5.18)	81.82 (±5.10)	87.73 (±3.59)	92.27 (±2.17)	95.45 (±1.84)	95.91 (±1.63)	97.27 (±0.79)

** *p < 0.01*,

* *p < 0.05*.

**Figure 7:**
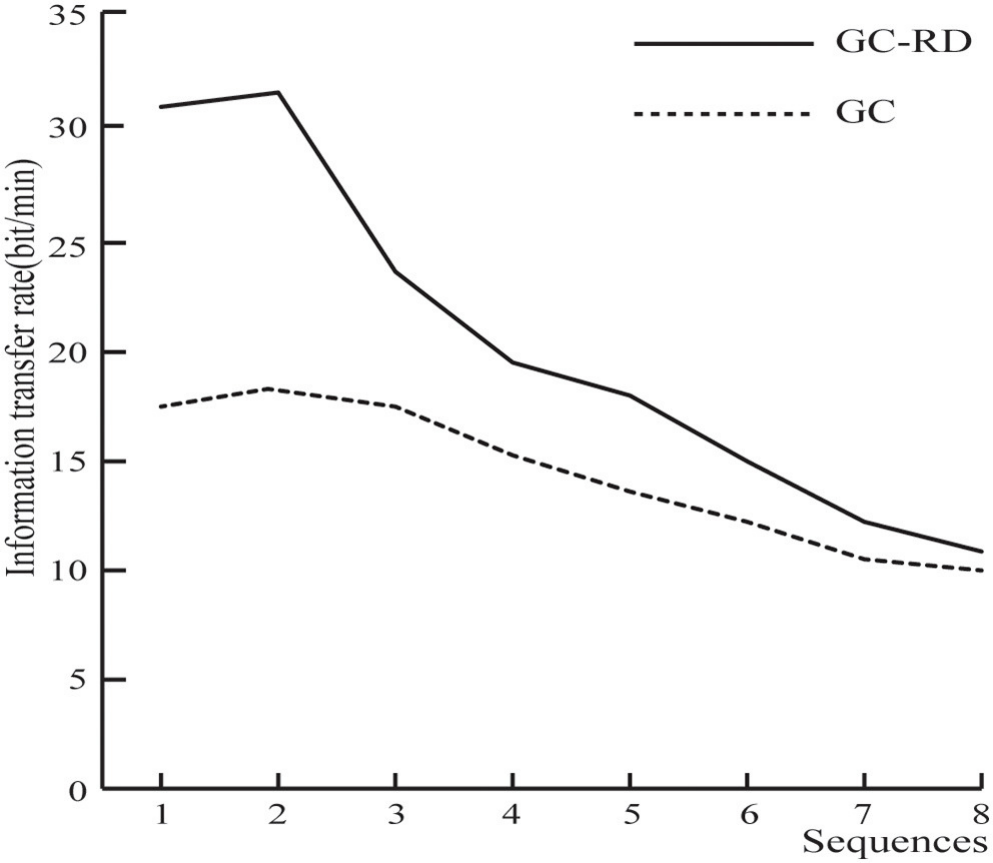
Mean information transfer rate (ITR) of 11 subjects in the GC-RD and GC spelling paradigms.

The mean ITR of the GC-RD spelling paradigm was higher than that of the GC paradigm for all sequences (Figure 7). The paired sample *t*-test for the ITR at each sequence between GC-RD and GC spelling paradigms was also conducted. Results showed that the differences were significant for sequences 1–7 (Table 2).

**Table 2:** Paired sample *t*-test results of information transmission rate (ITR) between the GC-RD and GC spelling paradigms at each sequence time.

	**Sequence**							
	1^**^	2^**^	3^**^	4^**^	5^*^	6^*^	7^*^	**8**
GC	17.85 (±3.37)	18.44 (±3.15)	17.81 (±2.50)	15.36 (±2.21)	13.72 (±1.61)	12.65 (±1.09)	11.18 (±0.85)	10.35 (±0.77)
GC-RD	30.80 (±5.94)	31.12 (±3.51)	23.96 (±2.46)	19.86 (±1.39)	17.16 (±0.74)	15.19 (±0.52)	13.13 (±0.42)	11.74 (±0.20)

** *p < 0.01*,

* *p < 0.05*.

## Discussion

Larger ERP amplitudes improve the performance of the P300 speller system. Our new GC-RD spelling paradigm is designed to enhance the attention of subjects on the target stimulus and to increase the visuospatial information. We compared the ERP amplitude, classification accuracy, and ITR between the GC-RD spelling paradigm and control paradigm (GC spelling paradigm).

A previous work has found that the P300 speller system's performance can be improved by enhancing the difference between target and nontarget trials (Jin et al., 2012). Therefore, we compared the waveforms (ERP_Target_ – ERP_Nontarget_) elicited in the GC-RD and GC spelling paradigms (Figure 5) and found two significant differences. The first was between 300 and 480 ms at the parietal area (Figure 5A), which is thought to be the P3a subcomponent of P300 (Polich, 2007). The P3a waveform usually occurs when subjects react to novel or small probability stimuli and is found at the frontal–central–parietal areas between 200 and 500 ms (Daffner et al., 2003; Berti, 2016; Li et al., 2019). The target stimuli in both the GC-RD and GC spelling paradigms elicited clear P3a components at the frontal–central–parietal areas, which is consistent with results of previous studies (Polich, 2007; Berti, 2016). The amplitude of P3a with a significant difference between GC-RD and GC spelling paradigms was found only at the parietal areas. Studies have suggested that the parietal area is activated when visual stimuli with spatial information are presented (Baumgartner et al., 2018) and when stimuli are located at the left and right sides of the screen (Wang et al., 2015), indicating that the parietal area is activated by visuospatial features. In a study of visuospatial information processing during attentional tasks, Abramov et al. (2017) found that target stimuli above or below the central fixation point elicited larger P300 amplitude at Pz (parietal area) than those without spatial information. At the same time, the analog ERP component was detected at Fz (frontal area) for stimuli with and without spatial information. This indicates that the increased P300 amplitude at Pz reflects the processing of visuospatial information about the target position during attentional tasks. In our GC-RD spelling paradigm, the red dot appeared randomly above or below the center of the green circle. This elicited a significantly increased P3a amplitude at parietal areas compared to the GC spelling paradigm. The increased P3a amplitude at the parietal area reflects brain processing of visuospatial information.

The second significant difference between GC and GC-RD was during 480–600 ms at the frontal–central–parietal areas (Figure 5B); this may be the P3b component, another subcomponent of P300. An early study showed that P3b appears in the frontal–central–parietal areas when attentional resources activate working memory, and the amplitude of P3b is influenced by the allocation of attentional resources to update working memory (Stevens, 1999), i.e., the P3b amplitude increases when cognitive demands are related to working memory (Li et al., 2019). Compared with the GC spelling paradigm, the positioning of the red dot in GC-RD imposed additional cognitive demands for the updating of working memory, which translated to significantly increased P3b amplitudes. Our findings are consistent with the study of Li et al. (2019), in which subjects were asked to pay attention not only to the number of target flashes but also to the color of the stimulus. Our GC-RD spelling paradigm deliberately added a red dot to the green circle to help subjects focus better on a small scope stimulus.

In addition, because the upper and lower positioning of the red dots in the green circle were random in the GC-RD paradigm, the probability of the target stimulus manifesting, decreased. Specifically, inclusion of the dots reduced the probability of the target stimulus manifesting from 2/13 (six rows or seven columns flashing) by 50%, to 1/13. Studies have consistently shown that the smaller the probability of the target stimulus appearing, the higher the level of the elicited P300 amplitude (Katayama and Polich, 1996). This is likely to be the reason that the GC-RD spelling paradigm elicited an increased P300 amplitude and improved the performance of the P300 speller system.

The ERP amplitude evoked by the GC-RD spelling paradigm was higher than that induced by the GC spelling paradigm. In addition the GC-RD spelling paradigm enhanced the difference between target and nontarget waveform and improved the classification accuracy (Jin et al., 2014). As expected, the average accuracies of the GC-RD spelling paradigm were higher than those of the GC spelling paradigm at each sequence (Figure 6). Moreover, there were significant differences in accuracy between the paradigms at all sequences (*p* < 0.05, Table 1) except sequence 8. Similarly, the ITR of the GC-RD spelling paradigm was significantly greater than that of the GC spelling paradigm at all sequences except sequence 8 (*p* < 0.05, Table 2). We also found that the improvements in ITR were even stronger at the first four sequences (*p* < 0.01), especially at sequence 2 (*p* < 0.0005). Thus, our results indicated that the GC-RD spelling paradigm significantly improved the performance of the P300 speller. Moreover, the results of accuracy and ITR further verify that the increased amplitude of waveforms (ERP_Target_ – ERP_Nontarget_) can improve the performance of the P300 spelling system. The ITR is an important statistical metric for the performance of the P300 speller system (Zhang et al., 2012). As we know, the ITR depends on both classification accuracy and the time to output a character based on the ITR calculation formula. The time to output a character is determined by the number of averaged sequences. As the number of averaged sequences reduces, the signal-to-noise ratio inevitably decreases and results in a decrease in classification accuracy. Therefore, classification accuracy and the number of averaged sequences must be weighed for obtaining a higher ITR (Li et al., 2015).

## Conclusion

This study investigated whether the new GC-RD spelling paradigm with small size and visuospatial information could improve the performance of the P300 speller. The results demonstrated that the GC-RD spelling paradigm enhanced the amplitude of the P300 potential and improved the classification accuracy and ITR at most sequence numbers compared with the GC spelling paradigm.

## Data Availability Statement

The raw data supporting the conclusions of this article will be made available by the authors, without undue reservation.

## Ethics Statement

The studies involving human participants were reviewed and approved by The Ethics Committee of Changchun University of Science and Technology. The patients/participants provided their written informed consent to participate in this study.

## Author Contributions

YW is responsible for experimental design. WZ is responsible for the implementation of the experiment. ZL is responsible for data analysis. QL is responsible for the overall idea of experimental design. All authors contributed to the article and approved the submitted version.

## Conflict of Interest

The authors declare that the research was conducted in the absence of any commercial or financial relationships that could be construed as a potential conflict of interest.

## References

[B1] Abramov D. M. Pontes M. Pontes A. T. Mourao-Junior C. A. Vieira J. Quero Cunha C.. (2017). Visuospatial information processing load and the ratio between parietal cue and target P3 amplitudes in the Attentional Network Test. Neurosci. Lett. 647, 91–96. 10.1016/j.neulet.2017.03.031 28336341

[B2] Aya R. Mihaly B. Piotr S. Felix G. Abdul S. Ivan V. (2018). Brain-computer interface spellers: a review. Brain Sci. 8, 57 10.3390/brainsci8040057 PMC5924393 29601538

[B3] Baumgartner H. M. Graulty C. J. Hillyard S. A. Pitts M. A. (2018). Does spatial attention modulate the earliest component of the visual evoked potential? Cogn. Neurosci. 9, 4–19. 10.1080/17588928.2017.1333490 28534668

[B4] Bernat E. Shevrin H. Snodgrass M. (2001). Subliminal visual oddball stimuli evoke a P300 component. Clin. Neurophysiol. 112, 159–171. 10.1016/S1388-2457(00)00445-4 11137675

[B5] Berti S. (2016). Switching attention within working memory is reflected in the P3a component of the human event-related brain potential. Front. Hum. Neurosci. 9, 701. 10.3389/fnhum.2015.00701 26779009 PMC4701918

[B6] Daffner K. R. Scinto L. F. M. Weitzman A. M. Faust R. Rentz D. M. Budson A. E.. (2003). Frontal and parietal components of a cerebral network mediating voluntary attention to novel events. J. Cogn. Neurosci. 15, 294–313. 10.1162/089892903321208213 12683359

[B7] Eriksen C. W. Yeh Y. Y. (1985). Allocation of attention in the visual field. J. Exp. Psychol. Hum. Percept. Perform. 11, 583–597. 10.1037/0096-1523.11.5.583 2932532

[B8] Farwell L. A. Donchin E. (1988). Talking off the top of your head: toward a mental prosthesis utilizing event-related brain potentials. Electroencephalogr. Clin. Neurophysiol. 70, 510–523. 10.1016/0013-4694(88)90149-6 2461285

[B9] Hugdahl K. Nordby H. (1994). Electrophysiological correlates to cued attentional shifts in the visual and auditory modalities. Behav. Biol. 62, 21–32. 10.1016/S0163-1047(05)80055-X 7945141

[B10] Jin J. Allison B. Z. Brunner C. Wang B. Wang X. Zhang J.. (2010). P300 Chinese input system based on Bayesian LDA. Biomed. Eng. 55, 5–18. 10.1515/bmt.2010.003 20128741

[B11] Jin J. Allison B. Z. Kaufmann T. Kubler A. Zhang Y. Wang X.. (2012). The changing face of P300 BCIs: a comparison of stimulus changes in a P300 BCI involving faces, emotion, and movement. PLoS ONE 7, e49688. 10.1371/journal.pone.0049688 23189154 PMC3506655

[B12] Jin J. Daly I. Zhang Y. Wang X. Cichocki A. (2014). An optimized ERP brain-computer interface based on facial expression changes. J. Neural Eng. 11, 036004. 10.1088/1741-2560/11/3/036004 24743165

[B13] Jin J. Sellers E. W. Zhou S. Zhang Y. Wang X. Cichocki A. (2015). A P300 brain-computer interface based on a modification of the mismatch negativity paradigm. Int. J. Neural Syst. 25, 1550011. 10.1142/S0129065715500112 25804352

[B14] Katayama J. Polich J. (1996). P300, probability, and the three-tone paradigm. Electroencephalogr. Clin. Neurophysiol. 100, 555–562. 10.1016/S0168-5597(96)95171-0 8980420

[B15] Kaufmann T. Schulz S. M. Grunzinger C. Kubler A. (2011). Flashing characters with famous faces improves ERP-based brain-computer interface performance. J. Neural Eng. 8, 056016. 10.1088/1741-2560/8/5/056016 21934188

[B16] Kubler A. Birbaumer N. (2008). Brain-computer interfaces and communication in paralysis: extinction of goal directed thinking in completely paralysed patients? Clin. Neurophysiol. 119, 2658–2666. 10.1016/j.clinph.2008.06.019 18824406 PMC2644824

[B17] Lakey C. E. Berry D. R. Sellers E. W. (2011). Manipulating attention via mindfulness induction improves P300-based brain-computer interface performance. J. Neural Eng. 8:025019. 10.1088/1741-2560/8/2/025019 21436516 PMC4429763

[B18] Lei X. Yang P. Yao D. (2009). An empirical bayesian framework for brain-computer interfaces. IEEE Trans. Neural Syst. Rehab. Eng. 17, 521–529. 10.1109/TNSRE.2009.2027705 19622442

[B19] Li Q. Liu S. Li J. Bai O. (2015). Use of a green familiar faces paradigm improves P300-speller brain-computer interface performance. PLoS ONE 10, e0130325. 10.1371/journal.pone.0130325 26087308 PMC4472698

[B20] Li Q. Lu Z. Gao N. Yang J. (2019). Optimizing the performance of the visual P300-speller through active mental tasks based on color distinction and modulation of task difficulty. Front. Hum. Neurosci. 13, 130. 10.3389/fnhum.2019.00130 31057381 PMC6478661

[B21] Lu Z. Li Q. Gao N. Yang J. Bai O. (2019). A novel audiovisual P300-speller paradigm based on cross-modal spatial and semantic congruence. Front. Neurosci. 13, 1040. 10.3389/fnins.2019.01040 31611770 PMC6777004

[B22] Mangun G. R. (1995). Neural mechanisms of visual selective attention. Psychophysiology 32, 4–18. 10.1111/j.1469-8986.1995.tb03400.x 7878167

[B23] Philip J. T. George S. T. (2020). Visual P300 mind-speller brain-computer interfaces: a walk through the recent developments with special focus on classification algorithms. Clin. EEG Neurosci. 51, 19–33. 10.1177/1550059419842753 30997842

[B24] Polich J. (2007). Updating P300: an integrative theory of P3a and P3b. Clin. Neurophysiol. 118, 2128–2148. 10.1016/j.clinph.2007.04.019 17573239 PMC2715154

[B25] Posner M. I. (1980). Orienting of attention. Q. J. Exp. Psychol. 32, 3–25. 10.1080/00335558008248231 7367577

[B26] Reza M. F. Ikoma K. Ito T. Ogawa T. Mano Y. (2007). N200 latency and P300 amplitude in depressed mood post-traumatic brain injury patients. Neuropsychol. Rehabil. 17, 723–734. 10.1080/09602010601082441 18030646

[B27] Rincover A. Ducharme J. M. (1987). Variables influencing stimulus overselectivity and “tunnel vision” in developmentally delayed children. Am. J. Ment. Defic. 91, 422–430. 3812612

[B28] Sellers E. W. Donchin E. (2006). A P300-based brain-computer interface: initial tests by ALS patients. Clin. Neurophysiol. 117, 538–548. 10.1016/j.clinph.2005.06.027 16461003

[B29] Stevens C. F. (1999). Memory: from mind to molecules. Nat. Med. 5, 1343–1344. 10.1038/70903 10581065

[B30] Sutton S. Braren M. Zubin J. John E. R. (1965). Evoked-potential correlates of stimulus uncertainty. Science 150, 1187–1188. 10.1126/science.150.3700.1187 5852977

[B31] Wang F. He Y. Pan J. Xie Q. Yu R. Zhang R. (2015). A novel audiovisual brain-computer interface and its application in awareness detection. Sci. Rep. 5, 9962 10.1038/srep12592 26123281 PMC4485169

[B32] Wolpaw J. R. Ramoser H. McFarland D. J. Pfurtscheller G. (1998). EEG-based communication: improved accuracy by response verification. IEEE Trans. Rehab. Eng. 6, 326–333. 10.1109/86.712231 9749910

[B33] Xia T. Qi Z. Shi J. Zhang M. Luo W. (2018). The early facilitative and late contextual specific effect of the color red on attentional processing. Front. Hum. Neurosci. 12, 224. 10.3389/fnhum.2018.00224 29950979 PMC6008538

[B34] Xiao X. Xu M. Jin J. Wang Y. Jung T. Ming D. (2019). Discriminative canonical pattern matching for single-trial classification of ERP components. IEEE Trans. Biomed. Eng. 67, 2266–2275. 10.1109/TBME.2019.2958641 31831401

[B35] Xu M. Han J. Wang Y. Jung T. Ming D. (2020). Implementing over 100 command codes for a high-speed hybrid brain-computer interface using concurrent P300 and SSVEP features. IEEE Trans. Biomed. Eng. 1, 1–10. 10.1109/TBME.2020.2975614 32149621

[B36] Xu M. Xiao X. Wang Y. Qi H. Jung T. Ming D. (2018). A brain-computer interface based on miniature-event-related potentials induced by very small lateral visual stimuli. IEEE Trans. Biomed. Eng. 65, 1166–1175. 10.1109/TBME.2018.2799661 29683431

[B37] Zhang Y. Zhao Q. Jin J. Wang X. Cichocki A. (2012). A novel BCI based on ERP components sensitive to configural processing of human faces. J. Neural Eng. 9, 026018. 10.1088/1741-2560/9/2/026018 22414683

[B38] Zhu J. Wang X. Q. He X. Hu Y. Y. Li F. Liu M. F.. (2019). Affective and cognitive empathy in pre-teachers with strong or weak professional identity: an ERP Study. Front. Hum. Neurosci. 13, 175. 10.3389/fnhum.2019.00175 31213999 PMC6555257

